# Mild Short‐Term Caloric Restriction Induces Coordinated Changes That Promote Lipid Deposition While Maintaining Thermogenesis Capacity in Interscapular Brown Adipose Tissue of Male Rats

**DOI:** 10.1096/fj.202503656R

**Published:** 2026-02-02

**Authors:** Giuliana Panico, Vincenzo Migliaccio, Gabriella Pinto, Anna Iliano, Gianluca Fasciolo, Martina Coletta, Paola Venditti, Maria Moreno, Angela Amoresano, Pieter de Lange, Lillà Lionetti, Assunta Lombardi

**Affiliations:** ^1^ Department of Biology University of Naples Federico II Napoli Italy; ^2^ Department of Chemistry and Biology “A. Zambelli” University of Salerno Fisciano Italy; ^3^ Department of Chemical Sciences University of Naples Federico II Napoli Italy; ^4^ Department of Science and Technology University of Sannio Benevento Italy; ^5^ Consorzio Interuniversitario Istituto Nazionale Biostrutture e Biosistemi—INBB Roma Italy; ^6^ Department of Environmental, Biological, and Pharmaceutical Sciences and Technologies University of Campania “Luigi Vanvitelli” Caserta Italy

**Keywords:** brown adipose tissue, caloric restriction, glycolysis, lipogenesis, mitochondria, thermogenesis

## Abstract

Calorie restriction (CR) without malnutrition improves metabolic health, slows the aging process, and prolongs longevity. Brown adipose tissue (BAT), which specializes in burning energy to produce heat, also contributes to metabolic health; however, the effects of CR on BAT function are only partially understood, and even less is known about those exerted by mild CR for a short period of time. We studied the multifaceted relationship between mild short‐term CR (15% applied for 2 weeks) and intrascapular BAT (iBAT) in rats and provided insights into the adaptations occurring in the tissue through multiple complementary approaches: histology, mitochondrial respirometry, comprehensive quantitative proteomics, and targeted metabolomics. Our data indicate that mild short‐term CR induces structural and metabolic adaptations in iBAT aimed at the deposition of triglycerides within the adipocyte, which appear paucilocular. CR increases the levels of enzymes involved in glycolysis while simultaneously reducing glycolytic intermediate metabolites. It also improves the levels of enzymes involved in de novo lipogenesis, in providing reducing power for lipid synthesis, and in lipid droplet dynamics. Although CR reduced mitochondrial iBAT content, tissue thermogenic potential was maintained, as it increased mitochondrial activity, compensating for their lower abundance. Overall, our data indicate that mild CR, within 2 weeks, affects BAT metabolism, inducing physiological adaptation that preserves its thermogenic capacity under conditions of energy deficit. These adaptations may represent a key mechanism underlying the systemic benefits of CR, offering new insights into the role of even mild and short‐term CR in maintaining overall health.

## Graphical abstract

Rats under mild caloric restriction (85% vs. ad libitum) for 2 weeks show metabolic and structural adaptations in iBAT. Caloric restriction increases glycolysis and triglyceride deposition in the tissue, leading to larger lipid droplets. It also reduces mitochondrial iBAT content, while maintaining iBAT thermogenic potential thanks to an increased mitochondrial activity compensating for their lower abundance.

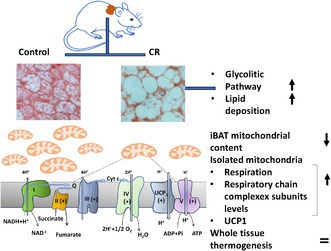

**Abbreviations**
1,3‐PG: 1,3‐Phosphoglycerate
2PG: 2‐Phosphoglycerate
3PG: 3‐Phosphoglycerate
6PG: 6‐Phosphogluconate Ru5P
6PGL: 6‐Phosphoglucono‐δ‐lactone
AcAc: acetoacetate
AcCoA: Acetyl‐CoA
ACN: acetonitrile
ANOVA: one‐way analysis of variance
BAT: Brown adipose tissue
BCAA: Branched‐chain amino acids
BSA: Bovine serum albumin
CD68: Cluster of Differentiation 68
CID: collision‐induced dissociation
CM: cytosolic mitochondria
CR: caloric restriction
DDA: Data Dependent Acquisition mode
DHAP: Dihydroxyacetone phosphate
DTT: dithiothreitol
ENO: Enolase
F1: 6BP Fructose‐1,6‐bisphosphate
F6P: Fructose‐6‐phosphate
FDR: false discovery rate
G1P: glucose‐1‐phosphate
G3P: Glycerol‐3‐phosphate
G6P: Glucose‐6‐phosphate
GAL: 3 Galectin 3
GAPD: glyceraldehyde 3 phosphate dehydrogenase
GDP: guanosine di‐phosphate
GLU: Glucose
Gly3P: Glyceraldehyde‐3‐phosphate
Gly‐3P: Glyceraldehyde‐3‐phosphate
HNE: hydroxynonenal
HSP‐60: heat shock protein 60
iBAT: interscapular BAT
KBs: Ketone bodies
KEGG: Kyoto Encyclopedia of Genes and Genomes
LC–MS/MS: liquid chromatography coupled with tandem mass spectrometry
LFQ: label‐free quantification
MPC2: mitochondrial pyruvate carrier 2
OXPHOS: oxidative phosphorylation complexes
PDH: pyruvate dehydrogenase
PDHK1 pyruvate: dehydrogenase Kinase
PEP: posterior error probability
PFKP: platelet type phosphofructokinase
PGAM1: phosphoglycerate mutase 1
PGLS: phosphogluconolactonase
PDM: peridrooplet mitochondria
PPP: pentose phosphate pathway
R5P: Ribulose‐5‐phosphate
S7P: Sedoheptulose‐7‐phosphate
SOD1: Superoxide dismutase 1
SOD2: Superoxide dismutase 2
TG: Triglycerides
UCP1: Uncoupling protein‐1
UDP‐Glc: Uridine Diphosphate Glucose
VDAC: voltage dependent anion channel
WAT: White adipose tissue
XuP: Xylulose‐5‐phosphate
α‐KG: alpha ketoglutarate

## 1 Introduction

Caloric restriction (CR) without malnutrition has become a promising intervention for improving metabolic health and extending lifespan. It is considered the most effective non‐pharmacological approach to counteract metabolic diseases, such as obesity and type‐2 diabetes, as well as reducing age‐related disorders like neurodegeneration and cancer [1, 2, 3, 4, 5]. Preclinical studies indicate that CR may produce its beneficial effects by potentially preventing or delaying key hallmarks of aging [4, 5] and human research has shown its effectiveness in reducing biomarkers of cellular senescence in healthy young to middle‐aged individuals without obesity [6].

One of the key features of CR is the significant reduction in adiposity; this effect may be important in the mechanisms of CR, given the endocrine role of white adipose tissue (WAT). In fact, extensive research on the impact of CR on WAT has shown that this intervention counteracts age‐related adiposity and metabolic issues by promoting weight loss and restoring tissue function, including lowering metabolic, hormonal, and inflammatory risk factors [7, 8].

In contrast to WAT, which mainly stores energy as triglycerides, brown adipose tissue (BAT) depots display a distinct profile characterized by a high number of mitochondria and the expression of uncoupling protein 1 (UCP1) [9]. The presence of UCP1 enables BAT to perform non‐shivering thermogenesis, primarily by uncoupling oxidative phosphorylation from ATP production, thereby dissipating energy as heat and providing a mechanism for maximizing mitochondrial substrate oxidation [10]. Brown adipocytes have unique features, including UCP1‐mediated proton leak processes, alongside the simultaneous occurrence of de novo fatty acid synthesis, fatty acid oxidation, and glycolysis [11, 12]. Indeed, upon activation, brown adipocytes increase lipolysis of stored triglycerides (TG) to support thermogenesis. Additionally, the cells utilize extracellular fatty acids originating from WAT lipolysis [13] or the metabolism of TG‐rich lipoproteins [14]. Within thermogenic adipocytes, both extracellular and intracellular fatty acids can be fully metabolized through mitochondrial β‐oxidation. At the same time, activation of glycolysis and de novo fatty acid synthesis can replenish part of the stored TG pool [11].

In BAT some mitochondria associate with lipid droplets (LD) (peridroplet mitochondria (PDM)), and they appear to possess characteristics distinct from cytoplasmic mitochondria (CM) [15]. PDM represents a separate mitochondrial population with unique structure and function that supports triglycerides synthesis. In fact, when compared to CM, PDM showed increased pyruvate oxidation, electron transport, and ATP synthesis capacities, while exhibiting a reduced beta oxidation capacity [15].

BAT exhibits the highest branched‐chain amino acids (BCAA) catabolism [16] and branched‐chain fatty acids synthesis fluxes [17]. Following cold exposure, BAT promotes systemic BCAA clearance in mice and humans [11]. In this context, a BAT‐specific defect in BCAA catabolism has been shown to attenuate systemic BCAA clearance, oxidation of BAT substrates, and thermogenesis, leading to diet‐induced obesity and glucose intolerance [18]. BCAA degradation is essential for the formation of branched‐chain alpha‐ketoacids, whose metabolism could lead to the formation of succinyl‐CoA from Propionyl‐CoA, and of acetyl‐CoA, thus feeding the Krebs cycle. Alternatively, BCAA can be addressed to the synthesis of branched‐chain fatty acids [17]. These unique characteristics establish BAT as a key player in regulating energy balance and metabolic homeostasis, and have prompted investigations into strategies to modulate its activity for therapeutic purposes [19] and for maintaining metabolic health, given evidence that its enhanced activity and functionality protect against obesity and diabetes [20, 21], as well as cardiovascular disorders [22, 23, 24]. Furthermore, a reduced amount of BAT is associated with obesity and glucose intolerance in humans [25]. Conversely, individuals with higher BAT activation tend to maintain lower body weight and experience healthier aging [26]. Therefore, BAT has the potential to support healthy longevity; however, to evaluate its real potential in humans, additional methods are needed beyond those currently used, which exploit the absorption of ^18^FG to determine its presence and abundance [27, 28, 29, 30]. CR, with its important rolein energy metabolism, emerges as a particularly interesting modulator of BAT function. However, the influence of CR on BAT physiology has been only partly understood, and research outcome may vary depending on the intensity and duration of the applied CR, the species of rodent considered, the animal's initial condition, particularly whether it is young or old, and its initial physical state, such as whether it is overweight or lean. In most studies CR administration varied between 30% and 50%, and was applied over extended periods of time. In this context, some studies on rats as animal model suggest that a long term CR shifts BAT function from an energy‐consuming system to an energy‐conserving one, promoting lipogenesis, accumulating TG, and reducing levels of proteins markers of mitochondrial activity and UCP1 expression [31]. Other studies indicate that CR increased mitochondrial respiration [32], and when applied for 12 months prevents the decline in mitochondrial function associated with aging, due to a lesser reduction in mitochondrial biogenesis [33]. Interestingly, the effects induced by CR appear to be dependent on the rodent model employed: studies using mice as animal model report that, in contrast to what has been observed in rat, CR reduced BAT lipid accumulation within the tissue [34]; in addition it prevents the aging induced‐enlargement of LD and the “whitening” of BAT [35, 36], In mice, in contrast to rats, CR has been reported to reduce mitochondrial respiration [37] while similarly reducing UCP1 levels [36].

Interestingly a recent study indicates that a metabolic shift occurs in mice BAT with varying levels of CR (10%–40% for 3 months), with changes in BAT metabolites even when caloric intake decreases by only 10% [38].

Although CR is vital for maintaining metabolic health and longevity, long‐term CR can be challenging to sustain and is often discontinued. In contrast, better adherence is expected with mild CR practised for a short period. Studying the effects of a mild CR in the short‐term could be important to understand how small changes can lead to metabolic adaptation without excessive risks, improving the quality of life and the prevention of diseases. Currently, our understanding of the complex relationship between mild short‐term CR and BAT is limited, and further research is necessary to gain insights into these interactions and tissue adaptations. Thus, our primary aim was to investigate the metabolic adaptation caused by a mild CR (15%) applied for a short period of time (2 weeks) on interscapular BAT (iBAT) physiology through multiple complementary methods, such as histology, mitochondrial respirometry (given the vital role of these organelles in tissue thermogenesis), along with western blot and a comprehensive quantitative proteomics approach using liquid chromatography with tandem mass spectrometry (LC–MS/MS), as well as a targeted metabolomics method. The results of the present work may be useful to further clarify the mechanisms through which BAT can contribute to the benefits of CR and in particular will allow us to evaluate whether these adaptive mechanisms are also activated by mild and short‐term caloric restriction.

## 2 Methods

### 2.1 Animals

In the present study, we used male Wistar rats obtained from Envigo RMS Srl, Udine, Italy, and housed in a temperature‐controlled room at 22°C ± 1°C under a 12:12‐h light–dark cycle.

At approximately 9 weeks of age, the rats were housed one per cage and randomly assigned to two different groups: control (C) and caloric restricted (CR), each consisting of 12 rats, with a similar mean body weight and with the body weights normally distributed within each group. During the first week, rats from both groups had free access to water and were fed ad libitum with a standard diet (4RF21, Mucedola, Italy); food intake of the two groups was daily monitored. Food intake was assessed by providing 180 g of chow to each rat around 10 a.m. and measuring the remaining chow and the spilled food collected from the cage after 24 h. After this initial week, the two groups displayed similar average food intake (about 22 g chow/day) and body weight (approximately 280 g), with rats from the C group continuing to be fed ad libitum with the standard diet for another 2 weeks, and the amount of food they consumed was determined daily. Rats from the CR group daily received an amount of chow equivalent to 85% of that consumed by the control group the day before for 2 weeks. The food was provided at about 11:00, and all food provided daily to the CR group was consumed by the rats.

For this study, we chose to house the rats at a standard room temperature (22°C), as it represents a mild cold stress for these rodents. Indeed, at the thermoneutral temperature, which for rats is approximately 28°C, BAT thermogenesis is at its lowest level, whereas at 4°C it is maximal [10]. Therefore, housing the rats at 22°C allows us to clearly observe both potential decreases and increases in BAT activity and changes in its morphology due to the treatment.

This study was conducted in accordance with the recommendations of the EU Directive 2010/63 on the Care and Use of Laboratory Animals. Every effort was made to minimize the suffering and pain of the rats. All animal protocols used throughout this study were approved by the Committee on the Ethics of Animal Experiments at the University of Naples Federico II and by the Italian Minister of Health (Authorization n° 776/2021‐PR).

At the end of the treatments, rats were anesthetized by i.p. administration of a single dose of sodium Tiopental (40 mg/Kg b.w.) and euthanized by decapitation. Tissues were excised and weighed. iBAT was immediately processed for homogenate preparation, mitochondrial isolation and histological analyses or frozen at −80°C for later analysis.

### 2.2 Histological Analyses

Samples of iBAT were fixed in Bouin solution (overnight at 4°C). The samples were dehydrated in ethanol, cleared, and embedded in paraffin blocks. The tissues were cut into serial 5‐μm‐thick sections; hematoxylin–eosin staining allowed morphological examination. To detect immunolocalization of such specific antigens, immunohistochemical staining was performed by using the Leica System kit (Novolynk Polymer Detection System). The procedure was performed according to the manufacturer. The primary antibody and dilutions used for analyses were VDAC (1:300 in PBS1X, ab GTX114187 GeneTex); HSP60 (1:300 in PBS1X, GTX110089 GeneTex) and UCP1 (1:500 in PBS1X, AB1426; Merck Life Science).

The sections were examined using a Nikon Eclipse 80i light microscope (Nikon Instruments, Milan, Italy). Images were captured with a Sony DS‐5 M camera linked to an ACT‐2 U image analyzer. Collagen molecules have a parallel structure, giving them natural birefringence. The use of picrosirius red, a specific histological dye for collagen, enhances the visibility of collagen fibers. When viewed under polarized light, tissues stained with picrosirius red show color variations that directly relate to the degree of collagen polymerization and their three‐dimensional organization.

### 2.3 In‐Solution Digestion for Proteomics Analysis

To obtain iBAT lysates, each tissue obtained from a single rat was homogenized in 10 volumes of RIPA buffer supplemented with protease inhibitor cocktails (Merck Life Science) using an Ultra‐Turrax homogenizer (T‐10 b, IKA Deutschland) with two 15‐s homogenization cycles to obtain iBAT homogenate. These latter were left on ice for 1 h, vortexed every 10 min, and then centrifuged at 17000 g for 30 min at 4°C. The resulting infranatant was collected and the protein concentrations were determined using the Bradford assay [39]. An aliquot of 100 μg of proteins from five different samples was pooled together to ensure equal contribution of each sample to the pool.

Proteins were precipitated by the addition of four volumes of ice‐cold acetone, followed by centrifugation at 10000 rpm for 10 min. The resulting pellets were air‐dried and resuspended in a denaturing buffer consisting of 6 M urea and 25 mM ammonium bicarbonate. Cysteine residues were reduced with 20 mM dithiothreitol (DTT) at 60°C for 1 h and subsequently alkylated with 40 mM iodoacetamide in the dark at room temperature for 1 h. Excess iodoacetamide was quenched with 20 mM DTT for an additional hour. Proteolytic digestion was carried out overnight (16–18 h) at 37 C using sequencing‐grade trypsin at an enzyme‐to‐substrate ratio of 1:50. The digestion was stopped by acidification with 1% formic acid before peptide desalting using C18 StageTips (three layers of 3 M Empore C18 discs). Tips were equilibrated and washed with 100 μL of 0.1% formic acid, and peptides were eluted sequentially with 50 μL of 50% acetonitrile (ACN) and 80% ACN, both containing 0.2% formic acid. The eluates were dried in a vacuum concentrator (SpeedVac) and reconstituted in 2% ACN with 0.2% formic acid for subsequent LC–MS/MS analysis.

### 2.4 LC–MS/MS Analysis

Peptides were analyzed on an LTQ Orbitrap XL (Thermo Fisher Scientific) coupled to a Proxeon nanoEasy‐II nanoLC system. Separation was performed on a C18 reverse‐phase capillary column (200 mm, 75 μm ID, 5 μm, 120 Å) with a flow rate of 250 nL/min. A linear gradient from 5% to 60% of buffer B (0.2% formic acid, 95% ACN) in buffer A (0.2% formic acid and 2% acetonitrile in MilliQ water) was applied over 200 min. The mass spectrometer was operated in Data‐Dependent Acquisition (DDA) mode over a mass range of 300–1800 m/z. In each scan, the five most intense precursor ions were selected for collision‐induced dissociation (CID), with a dynamic exclusion window of 40 s applied.

### 2.5 Bioinformatics Processing

Raw mass spectrometry data were analyzed using the Andromeda search engine embedded in the MaxQuant software suite (version 1.6.8.0) [40]. Peptide and protein identification was performed against the *Rattus norvegicus* reference proteome, retrieved from the UniProt database. Search parameters included trypsin specificity allowing up to three missed cleavages, carbamidomethylation of cysteine as a fixed modification, and methionine oxidation, N‐terminal pyro‐glutamate formation (from glutamine), and β‐hydroxybutyrylation of lysine as variable modifications. The minimum peptide length was set to six amino acids, and at least one unique or razor peptide was required for protein identification. Mass tolerances were set to 10 ppm for precursor ions and ±0.02 Da for the fragment ions. A false discovery rate (FDR) threshold of 1% was applied at both the peptide and protein levels.

Quantitative data analysis was performed using Perseus software (version 1.6.8.0) [41]. Technical replicates were merged, and entries corresponding to potential contaminants, reverse identifications, or proteins detected solely by site were excluded from further analysis. Label‐free quantification (LFQ) intensity values were log_2_‐transformed, and proteins with at least two valid measurements across experimental conditions were retained. Missing values were imputed using a normal distribution‐based approach (width = 0.3; downshift = 1.8). The resulting dataset included 488 proteins (see File S1), annotated with UniProt accession numbers, gene and protein names, identification scores, and sequence coverage. Differential protein expression was determined based on LFQ intensity ratios between CR and control conditions, with thresholds set at fold change > 1.3 for upregulation and < 0.77 for downregulation.

The STRING software was used to integrate all known and predicted associations between regulated proteins, including both physical interactions and functional associations [42]. A full STRING network (the edges indicate both functional and physical protein associations), a medium confidence of 0.4, and k‐means clustering (the network is clustered to a specified number of clusters) were set.

Kyoto Encyclopedia of Genes and Genomes (KEGG) enrichments were obtained by String and by the KEGG database [43] by converting Uniprot id into KEGG annotation (genome annotation database).

### 2.6 Metabolomics Analysis

#### 2.6.1 Sample Preparation

iBAT lysate was prepared in RIPA buffer as described above. Tissue lysates were individually subjected to protein precipitation and metabolite extraction by the addition of four volumes of an organic solvent mixture (1:1, v/v, acetonitrile: methanol). The resulting supernatants were subsequently diluted 1:10 with an aqueous solution containing 0.1% formic acid and 5 mM ammonium formate. Metabolite extracts were then analyzed by liquid chromatography–tandem mass spectrometry (LC–MS/MS) using Multiple Reaction Monitoring ion mode for targeted analyses.

#### 2.6.2 LC‐MRM/MS Analysis

The analyses were performed using Sciex 5500 QTrap mass spectrometry system equipped with exion LCTM. Chromatographic separation was performed by a Kinetex C18 column (5 μm particle size, 100 Å pore size, 100 × 2.1 mm; Phenomenex) at 35 C. Separation was carried out over an 8‐min run at a flow rate of 0.2 mL/min, employing a binary gradient from 2% B to 98% B. Mobile phase A was composed of ultrapure water supplemented with 0.1% (v/v) formic acid and 5 mM ammonium formate, whereas mobile phase B consisted of acetonitrile containing 0.1% (v/v) formic acid. Electrospray Ionization (ESI) was carried out in positive ion mode for amino acid detection and in negative ion mode for organic acids. The ESI source was operated under the following conditions: curtain gas (CUR) at 20 psi, collision gas (CAD) set to medium, ion spray voltage (IS) at 5.5 kV, and a source temperature (TEM) of 500°C.

Instrumental parameters for the MRM method, including precursor and product ion m/z values, collision energies, and dwell times, were configured according to previously published protocols [44, 45]. Data acquisition files (.wiff) were processed using Skyline software [46]. It was employed to verify the co‐elution of MRM transitions corresponding to each target analyte and to extract chromatographic peak areas for quantitative assessment across biological samples.

### 2.7 Determination of Mitochondrial Respiration Rate in Whole BAT Homogenate and Isolated Mitochondria

Immediately after excising the tissue, iBAT free of any visible WAT contamination was immersed in an ice‐cold isolation buffer (220 mM mannitol, 70 mM sucrose, 20 mM Tris–HCl, 1 mM EDTA, 5 mM EGTA, and 0,5% fatty acid‐free bovine serum albumin BSA, pH 7.4). The tissue was homogenized in 10 volumes of isolation buffer using a Potter‐Elvehjem homogenizer and centrifuged at 10.000 × *g* for 10 min at 4°C. The floating fat was removed, and the pellet was re‐suspended in the supernatant. The fat‐free homogenate was stored at 4°C until required for respiratory measurements.

The initial centrifugation of the homogenate at 10.000 × *g* separates PDM from LD [15, 47], ensuring that most of them are not lost during the removal of floating fat.

To isolate mitochondria, BAT was homogenized in 10 volumes of isolation buffer, and the entire homogenate was centrifuged at 10.000 × *g*. The supernatant was discarded, and the resulting pellet was re‐suspended in the original volume and centrifuged at 700 × *g*. The resulting supernatant was then centrifuged at 8.000 × *g* to pellet the mitochondria. Mitochondria were washed twice and re‐suspended in a minimal volume of isolation medium lacking BSA, kept on ice until use. Since the protocol used for mitochondria isolation includes an initial high‐speed centrifugation step (10.000) that strip PDM form LD [15, 47], the isolated mitochondrial enriched fraction obtained resulted in a mixed population of CM and PDM.

The mitochondrial respiration rate was evaluated polarographically using a Clark‐type electrode (Hansatech Instruments Ltd., United Kingdom). Fat‐deprived homogenate or isolated mitochondria were incubated in a respiration medium composed of 80 mM KCl, 50 mM HEPES (pH 7.0), 1 mM EGTA, 5 mM K_2_HPO_4_, and 0.5% fatty acid‐free BSA (w/v). and maintained at 37°C. Respiration was started by adding one of the following substrates: Succinate + rotenone (10 mM, 3,7 μM), Pyruvate + malate (5 mM, 2 mM). Successively, guanosine diphosphate (GDP) (2 mM) was added to the respiration medium.

To evaluate the ability of mitochondria to use Glycerol‐3‐phosphate (G3P) as a substrate for respiration, mitochondria were incubated in the above respiration medium, and respiration was started by adding G3P (5 mM) to the respiration medium.

All reagents used for mitochondrial isolation and respiration rate detection were from Merck Life Science S.r.l.

### 2.8 Western Blot

Western Blot was performed on iBAT lysate and mitochondrial lysate samples; iBAT lysate samples were prepared as described above. To obtain mitochondrial lysates, the isolated mitochondria, prepared as described above, were diluted in RIPA buffer (supplemented with antiprotease cocktails), left on ice for 1 h, vortexed every 10 min, and then centrifuged at 17000 *g* for 30 min at 4°C. The resulting supernatant was collected.

Protein concentration was determined using Bio‐Rad's DC assay (Bio‐Rad Laboratories, Hercules, CA).

Primary antibodies used throughout the study were the following: anti‐uncoupling protein 1 (UCP1, AB1426; Merck Life Science), a cocktail of antibody used to detect CI‐NDUF88, CII‐SDHB, CIII‐UQCRC2, CIV‐MTCO1, and CV‐ATP VA subunits of oxidative phosphorylation complexes (Total OXPHOS Rodent WB Antibody Cocktail, ab110413, Abcam, Cambridge UK). Anti‐voltage dependent anion channel (VDAC, ab GTX114187 GeneTex Inc. USA), anti‐heat shock protein 60 (HSP‐60, GTX110089 GeneTex Inc.), anti‐vinculin (PA5‐80216 Invitrogen, Thermofisher Scientific, Italy).

Anti pyruvate dehydrogenase (PDH 3205 Cell Signaling Technology, Danvers Massachusetts, USA), anti hexokinase II (Hexokinase II 2024 Cell Signaling Technology), anti platelet type phosphofructokinase (PFKP 8164 Cell Signaling Technology), anti glyceraldehyde 3 phosphate dehydrogenase (GAPDH 5174 Cell Signaling Technology), anti phosphoglycerate mutase 1 (PGAM1 12 098 Cell Signaling Technology), anti pyruvate dehydrogenase Kinase (PDHK1 3820 Cell Signaling Technology), anti Enolase (ENO 3810, Cell Signaling Technology), anti mitochondrial pyruvate carrier 2 (MPC2 46 141 Cell Signaling Technology), anti‐Superoxide dismutase 1 (SOD1 4266 Cell Signaling Technology), anti Superoxide dismutase‐2 (SOD2 ab13533 Abcam, Cambridge), anti hydroxynonenal adduct (HNE adduct ab46545 Abcam); Cluster of Differentiation 68 (CD68, sc‐59 103 SantaCruz Biotechnology, Inc USA); Galectin 3 (GAL3, sc‐20 157 SantaCruz Biotechnology, Inc).

The primary antibodies were diluted 1:1000, with the exception of total OXPHOS, CD68 and galectin that were diluted 1:500.

### 2.9 Lipid Hydroperoxide Levels and Susceptibility of BAT to Oxidative Insult

BAT lipid hydroperoxides (Hps) levels were measured in tissue homogenates (0.01 mg proteins) by assessing the decrease in NADPH absorption at 340 nm, which results from the oxidation of NADPH by the glutathione peroxidase–glutathione reductase system in the presence of GSH [48], using a multi‐plate reader (Synergy HTX Multimode Microplate Reader, BioTek).

The susceptibility of BAT homogenates to an in vitro oxidative stress insult was assessed as reported by Fasciolo et al. (2023) [49]. The changes in Hps levels induced by treatment of the BAT homogenate (0.01 mg protein) with Fe and ascorbate (Fe/As 100/1000 μM) for 10 min at room temperature were assessed. The reaction was stopped by adding 0.2% 2.6‐di‐t‐butyl‐p‐cresol. The hydroperoxide levels were evaluated as described above.

Reagents used were from Merck Life Science.

### 2.10 Cytosolic and Mitochondrial Glycerol‐3‐Phosphate Dehydrogenase Activities

To evaluate the activity of cytoplasmic glycerol 3‐phosphate dehydrogenase (GPD1), BAT homogenate (0.01 mg of protein) was incubated with 4 mM NAD+ and 5 mM glycerol‐3‐phosphate (G3P), and the production of NADH was monitored at 340 nm using a multiplate reader (Synergy HTX Multimode Microplate Reader, BioTek) [50]. One unit of enzyme activity is defined as 1 μmol NAD^+^ reduced per minute per mg of protein.

The Activity of mitochondrial glycerol‐3‐phosphate dehydrogenase (GPD2) was assayed as described in Pecinovà et al. (2020) [51] in 0,01 mg of proteins as glycerol‐3‐phosphate: 2,6‐dichlorophenolindophenol oxidoreductase. The assay medium contained 50 mM KCl, 10 mM Tris–HCl, 1 mM EDTA, 1 mg/mL BSA and 1 mM KCN, pH 7.4. The reaction was started by adding 25 mM G3P and 100 μM DCPIP and the change of the absorbance was monitored for 60 s at 30°C at 610 nm (ε610 = 20.1/mM/cm). The Enzyme activity was expressed as nmol min^−1^ × mg protein^−1^ and measured with a multiplate reader (Synergy HTX Multimode Microplate Reader, BioTek).

Reagents used were from Merck life Science S.r.l.

### 2.11 Statistical Analysis

Data were analyzed with the GraphPad Prism 6 software system. A two‐tailed unpaired *t*‐test was used to determine the statistical significance of differences between the two experimental groups. Differences were considered statistically significant at *p* < 0.05.

## 3 Results

### 3.1 CR Reduces Body Weight Gain and Affects iBAT Morphology

**FIGURE 1:**
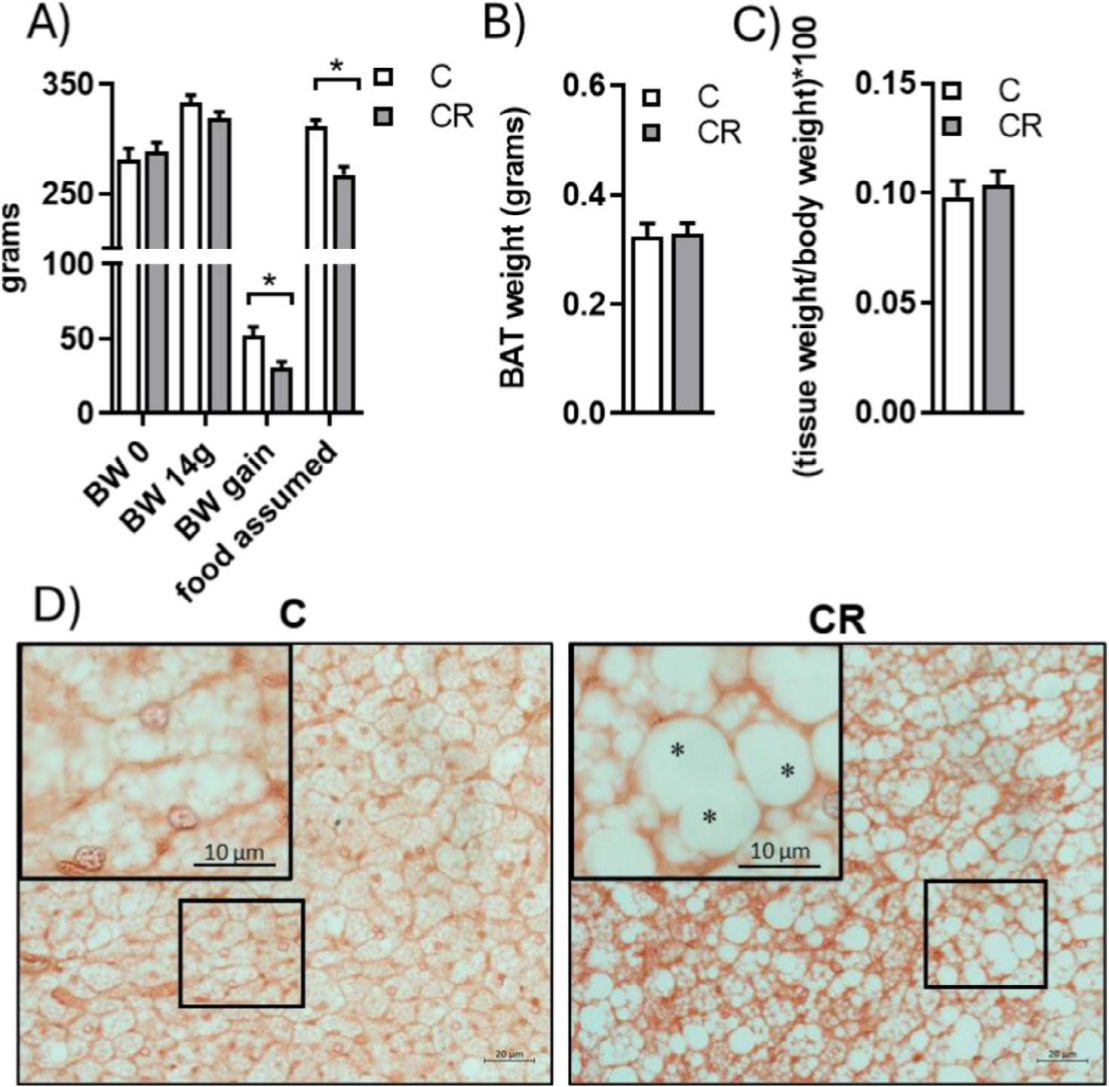
Effect of mild short‐term CR on rats body weight gain, energy assumption iBAT weight (A‐C) and iBAT morphology (D). Histological Images are representative of iBAT sections. Hematoxylin and eosin staining showed different cellular shapes between the two groups within BAT parenchyma, and a different size of lipid droplets inside the multilocular adipocytes. CR group showed a lot of paucilocular adipose cells (*). Inset represent high magnification of the frame areas. Magnifications used: 40X or 100X (insert); scale bars applied: 20 μm and 10 μm (insert). In the panels A‐C, histograms represent the mean ± SEM of 12 different rats. *p < 0.05 versus C.

At the start of the caloric restriction treatments, the rats had similar body weights (279 ± 8 g and 286 ± 7 g in C and CR, respectively *n* = 12) and after 2 weeks of treatment the CR groups showed a reduction in body weight gain (50 ± 5 and 33 ± 3 g in C and CR, respectively). CR did not affect BAT wet weight and its contribution to the total body weight (Figure 1A).

iBAT morphology showed changes in lipid droplet size and organization in cells. In fact, hematoxylin and eosin stain revealed that the CR animal group had an increase in lipid droplet diameter compared to the C group, indicating fat accumulation in the tissue. In CR group, many cells underwent morphological changes, appearing paucilocular compared to the multilocular adipocytes in the C group (Figure 1B).

### 3.2 CR Affects the Whole iBAT and Mitochondrial Proteomic Pattern

**FIGURE 2:**
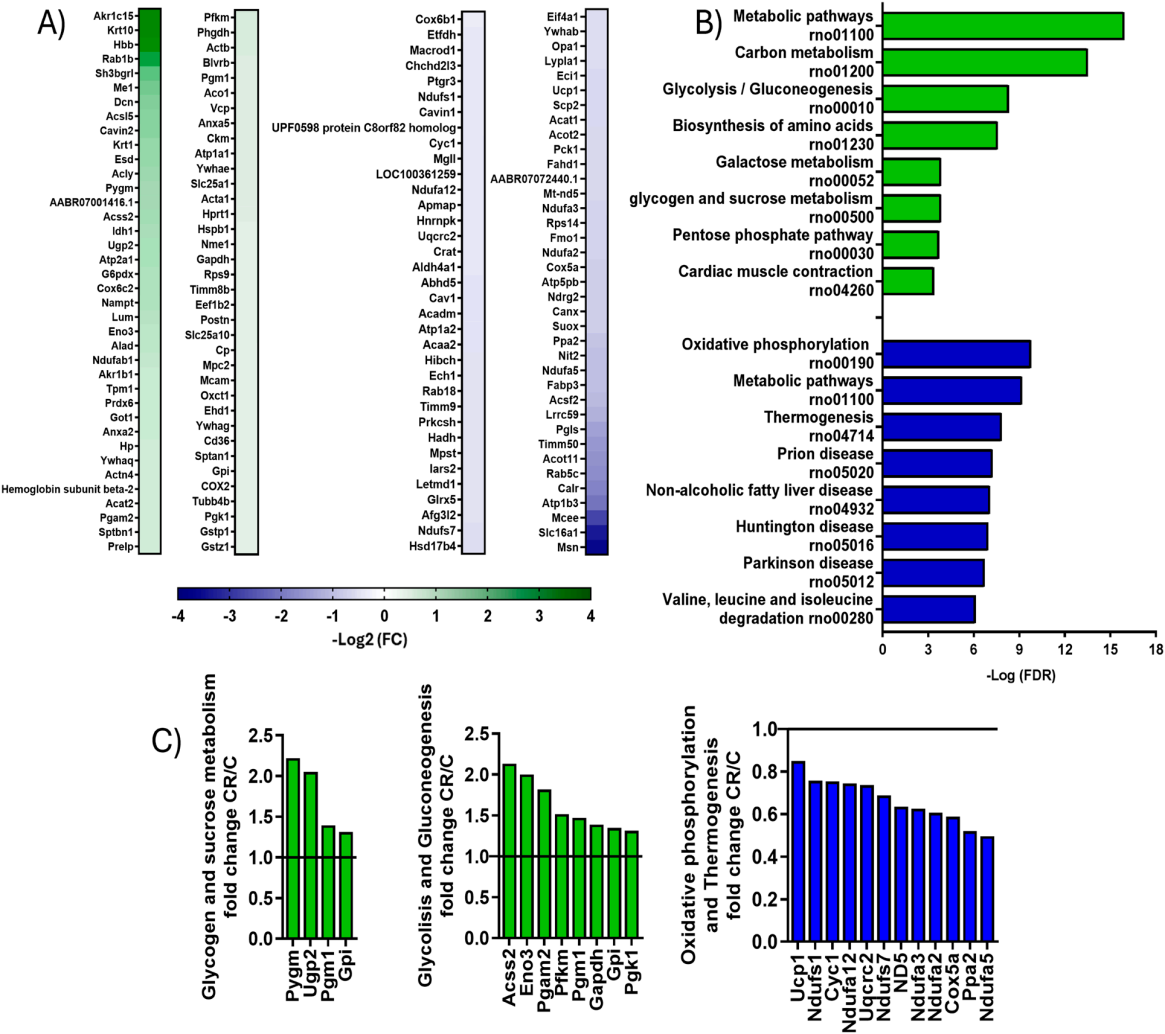
Effect of mild short‐term CR on the BAT proteome. (A) Heatmap depicting proteins dysregulated by CR of at least 1.3‐ fold. Data are reported as Log_2_ (fold change, FC). The names of the genes of the dysregulated protein are reported. (B) KEGG pathway analysis of the top KEGG enriched gene pathway, in green are reported the upregulated pathways, in blue the downregulated ones. (C) Relative abundance of proteins involved in pathways up and down‐regulated by CR. The continue line represents the values relative to the control rats set to 1. Each value is the result of two technical replicates obtained by a pool of 5 rats for each condition.

We performed a proteomic analysis of iBAT to examine the molecular changes caused by treatments. iBAT proteins underwent traditional in‐solution digestion for a large‐scale shotgun bottom‐up approach using Liquid Chromatography–Tandem Mass Spectrometry LC–MS/MS analysis. The full list of identified proteins, UniProt ID, gene name, sequence coverage [%], score, and the ratios of CR/C LFQ intensities are provided in (Supporting Information S1). In the entire iBAT lysate, we identified 486 proteins, with 30% being dysregulated by at least 1.3‐fold following CR treatment; of these, half were upregulated and half downregulated (Figure 2A).

The proteins upregulated more than 2‐fold by CR include Aldo‐keto reductase family 1, member C15 (*Akr1c15*); Keratin, type I cytoskeletal 10 (*Krt10*); member RAS oncogene family Rab1b (*RAB1B*); SH3 domain‐binding glutamic acid‐rich‐like protein (*Sh3bgrl1*); Malic enzyme (*Me1*); Decorin (*Dcn*); Long‐chain‐fatty‐acid‐CoA ligase (*Acsl5*); Caveolae‐associated protein 2 (*Cavin2*); Keratin, type II cytoskeletal 1 (*Krt1*); S‐formylglutathione hydrolase (*Esd*); ATP‐citrate synthase (*Acly*); Alpha‐1,4 glucan phosphorylase (*Pygm*); IF rod domain‐containing protein (AABR07001416.1); Acetyl‐coenzyme A synthetase, cytoplasmic (*Acss2*); Isocitrate dehydrogenase [NADP] cytoplasmic (*Idh1*); UTP‐glucose‐1‐phosphate uridylyltransferase (*Ugp2*); Calcium‐transporting ATPase (*Atp2a1*); Glucose‐6‐phosphate 1‐dehydrogenase (*G6pdx*); Cytochrome c oxidase subunit 6C‐2 (*Cox6c2*) (Figure 2A). The proteins that were downregulated include: Omega‐amidase NIT2 (*Nit2*); NADH dehydrogenase [ubiquinone] 1 alpha subcomplex Ndufa5; Fatty acid‐binding protein (*Fabp3*); Mitochondrial Medium‐chain acyl‐CoA ligase (*Acsf2*); Leucine‐rich repeat‐containing protein 59 (*Lrrc59*); 6‐phosphogluconolactonase (*Pgls*); Mitochondrial import inner membrane translocase subunit (*Timm50*); Acyl‐CoA thioesterase 11 (*Acot11*); RAB5C, member RAS oncogene family (*Rab5c*); Calreticulin (*Calr*); Sodium/potassium‐transporting ATPase subunit beta‐3 (*Atp1b3*); Methylmalonyl CoA epimerase (*Mcee*); Monocarboxylate transporter 1 (*Slc16a1*); Moesin (*Msn*).

To gain further insights into the processes and pathways regulated by CR, KEGG pathway analysis was performed, focusing on proteins dysregulated by at least 1.3‐fold due to CR (Figure 2B and File S1).

When considering the up‐regulated proteins, the primarily enriched pathways are Metabolic pathways, Carbon metabolism, Glycolysis/Gluconeogenesis, Biosynthesis of amino acids, Galactose metabolism, Glycogen metabolism, Pentose phosphate pathway, and Cardiac muscle contraction (Figure 2B green bars).

The pathways that are principally enriched in proteins down‐regulated by CR are Oxidative phosphorylation, Metabolic pathways, Thermogenesis, Prion disease, Non‐alcoholic fatty liver disease, Huntington disease, Parkinson's disease, Valine, leucine, and isoleucine degradation (Figure 2B, blue bars).

Proteins involved in Glycolysis/Gluconeogenesis, Glycogen metabolism, Oxidative phosphorylation, and Thermogenesis dysregulated by CR are also reported in Figure 2C.

### 3.3 CR Influences Levels of Enzymes and Metabolites Involved in Glucose Metabolism Pathways

As described above, according to KEGG enrichment analyses, some proteins up‐regulated by CR belong to the network of glycogen metabolism and the glycolytic pathway (Figure 2B,C).

**FIGURE 3:**
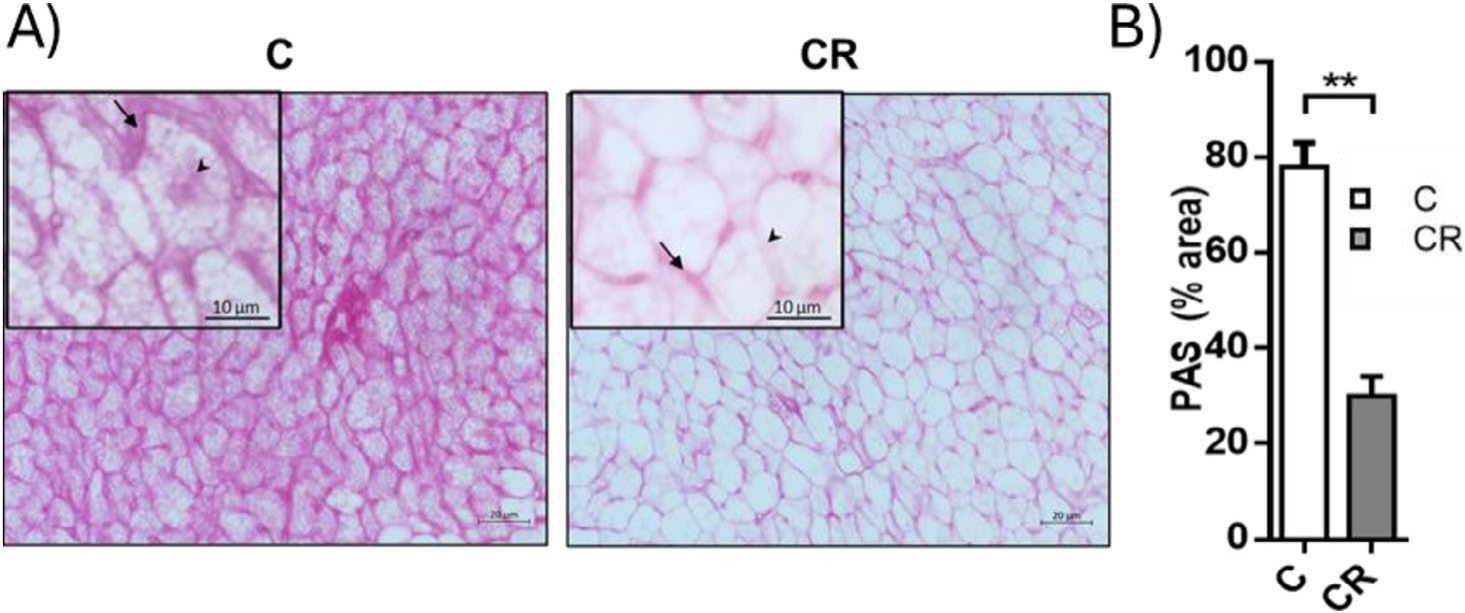
Effect of mild short‐term CR on glycogen levels. (A) Representative iBAT sections stained with periodic acid shiff (PAS); insert represents high magnification of the frame area. Arrows indicate extracellular stained regions, whereas arrowheads indicate PAS‐positive intracellular areas. Magnifications used: 40X or 100X (insert); scale bars applied: 20 μm and 10 μm (insert). (B) Quantification of PAS staining. Histograms represent the mean ± SEM of 4 different rats. *p < 0.05 versus C.

Concerning proteins involved in glycogen metabolism and affected by mild short‐term CR, they participated in both glycogenolysis and glycogenesis. Indeed, both Uridine diphosphate‐glucose pyrophosphorylase (UGP2), a key enzyme in glycogenesis, and glycogen phosphorylase (PYGM), a key enzyme involved in glycogenolysis, were enhanced by about 2‐fold. In addition, CR also increased the levels of Phosphoglucomutase 1 (PGM1), which catalyzes the reversible isomerization of alpha‐D‐glucose 1‐phosphate to alpha‐D‐glucose 6‐P (Figure 2A,C). Histochemical staining of iBAT sections with periodic acid Schiff (PAS), which visualizes molecules with a high percentage of carbohydrate content, such as glycogen, indicates a reduction in staining intensity of iBAT sections from CR treated rats (Figure 3). These data indicate a lower glycogen accumulation within the tissue and suggest the predominance of glycogenolysis over glycogenesis in BAT of CR treated rats.

**FIGURE 4:**
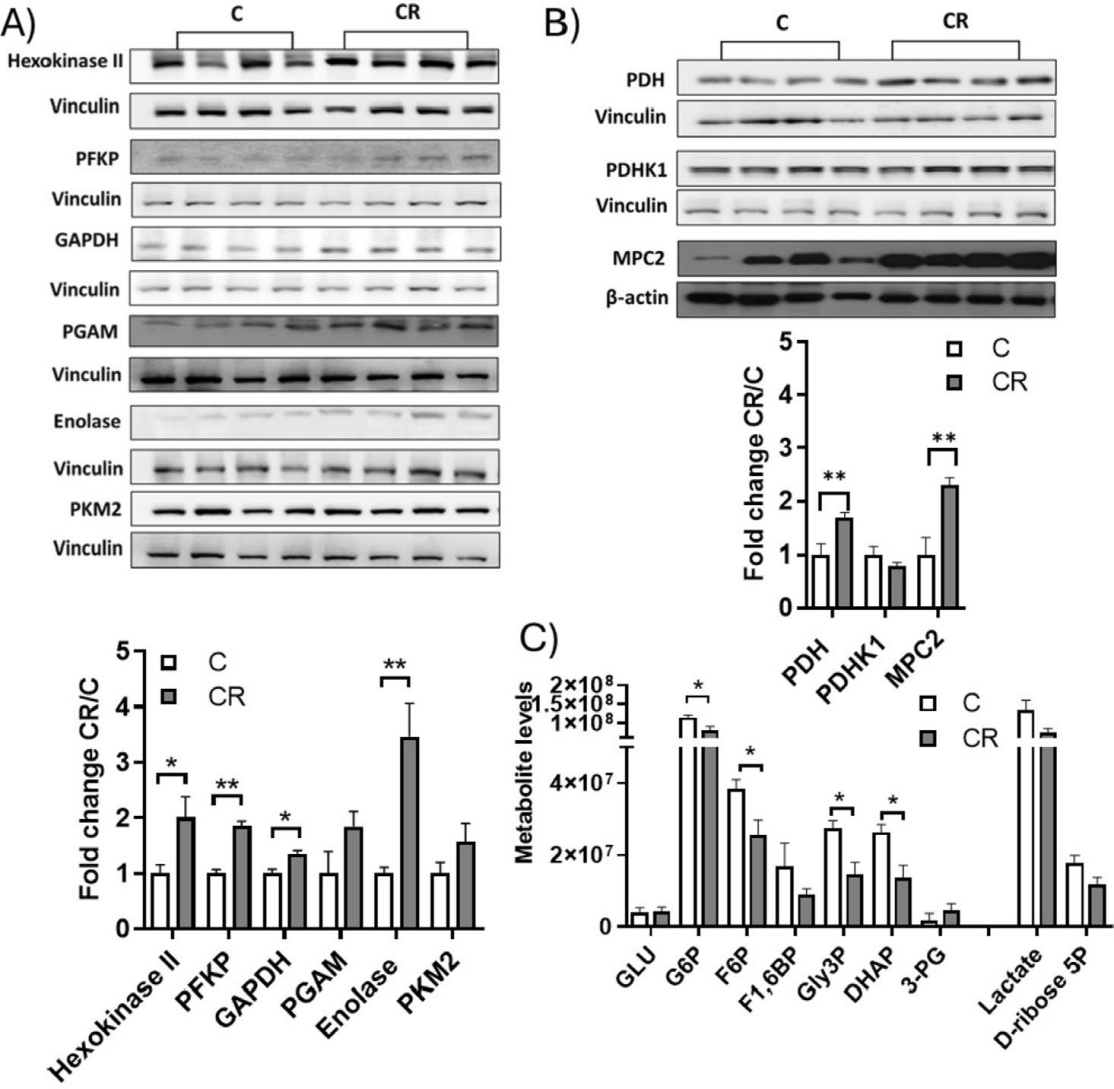
Effect of mild short‐term CR on glycolysis pathway. (A) Western Blots of proteins involved in glycolysis/gluconeogenesis pathways and pyruvate metabolism. Vinculin or β actin was used as the loading control. (B) Histograms represent quantification of the western blot data, which were normalized to the value obtained for C animals, set as 1. Each lane contained 25 μg of protein of total iBAT lysate from a single rat. (C) Levels of glycolysis intermediates obtained with targeted metabolomics. Values are represented as mean ± SEM of at least 4 different rats *p < 0.05 versus C **p < 0.01 versus C. Abbreviation used in the panel (C) are: GLU: Glucose; G6P: Glucose‐6‐phosphate, F6P: Fructose‐6‐phosphate, F1,6BP: Fructose‐1,6‐bisphosphate, Gly‐3P: Glyceraldehyde‐3‐phosphate DHAP: Dihydroxyacetone phosphate, 3PG: 3‐Phosphoglycerate.

As shown in Figure 2B, KEGG enrichment analysis indicated that CR elevates the protein levels of enzymes involved in the glycolytic pathway. This was verified by Western blot analysis, which revealed increased levels of hexokinase, phosphofructokinase (PFKP), enolase, and glyceraldehyde dehydrogenase (GAPDH) (Figure 4A,B).

In addition, the levels of mitochondrial pyruvate carrier isoform 2 (MPC2) are increased by CR as shown by Western blot (Figure 4A,B) and proteomic data (see also File S1). CR also enhanced pyruvate dehydrogenase (PDH) levels, while not significantly affecting pyruvate dehydrogenase kinase (PDK1) (Figure 4A,B).

To further examine the impact of CR on the glycolytic pathway, through target metabolomics we measured the levels of certain intermediate metabolites (Figure 4C). CR did not influence glucose levels in iBAT, but it decreased the levels of glucose‐6‐phosphate, fructose‐6‐phosphate, fructose‐1,6‐bisphosphate, and dihydroxyacetone phosphate. Pyruvate levels were below the detection limits of the equipment.

Interestingly, since CR oppositely regulates two proteins involved in the first and second steps of the pentose phosphate pathway (PPP), such as glucose‐6‐phosphate dehydrogenase (G6PD), which was upregulated, and 6‐phosphogluconolactonase (PGLS), which was downregulated (Figure 2A), we measured the levels of D‐ribose 6‐phosphate, a product of the PPP pathway. D‐ribose 6‐phosphate tended to be reduced in iBAT from CR rats; however, the differences did not reach statistical significance (Figure 4C).

### 3.4 CR Enhances Mitochondrial Respiration When Detected in Isolated Mitochondria but Not in Total Tissue Lysate

Given the evidence that iBAT mitochondria are the principal site of thermogenesis, we investigated the ability of CR to affect their functionality by integrating western blot, proteomics, immunohistochemistry, and respirometry data.

**FIGURE 5:**
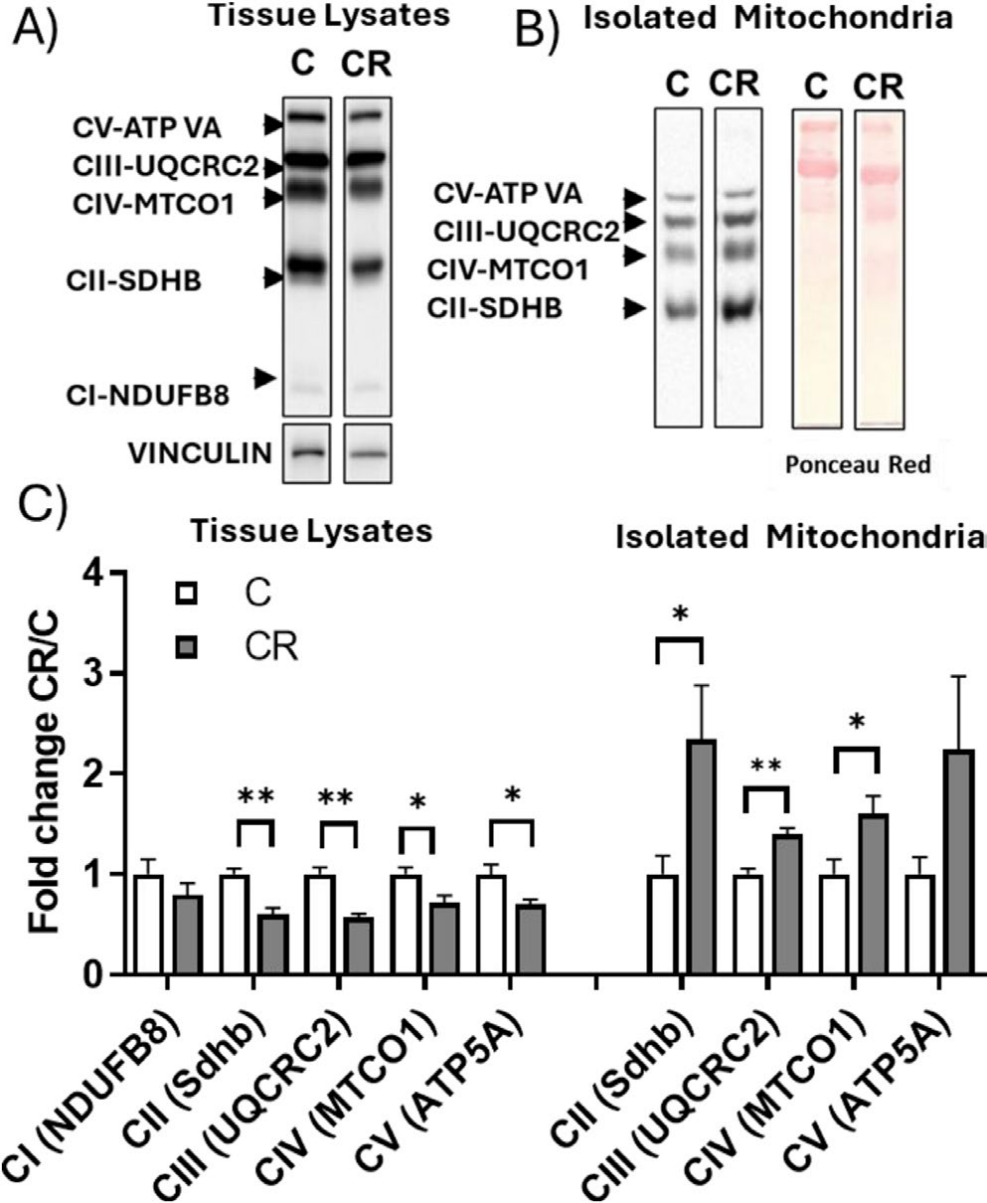
Representative Western blots of respiratory complexes subunits (CII‐SDHB, CIII‐UQCRC2, CIV‐MTCO1, and CV‐ATPVA), detected in the whole iBAT lysate (A) and isolated mitochondria (B). Vinculin or Ponceau staining was used as a loading control. Each lane contained 25 μg of protein from a single rat. Histograms represent quantification of the western blot data, which were normalized to the value obtained for C animals, set as 1 (C). Values represent the mean ± SEM of 6 different rats. *p < 0.05, **p < 0.01 versus C.

KEGG analysis of dysregulated proteins detected by the proteomic approach indicated that oxidative phosphorylation is one of the mainly enriched pathways in proteins down‐regulated by CR (Figure 2C). Consistent with the proteomic data, Western blot analysis of specific subunits belonging to the five mitochondrial respiratory complexes, that is, NDUFB8 (complex I), SDHB (complex II), UQCRC2 (complex III), MTCO1 (complex IV), and ATP5A (complex V), showed that these proteins were reduced in iBAT lysate (Figure 5A,C).

Interestingly, when detected in the isolated mitochondria, the respiratory complexes' subunits mentioned above were all upregulated by CR (Figure 5B,C).

**FIGURE 6:**
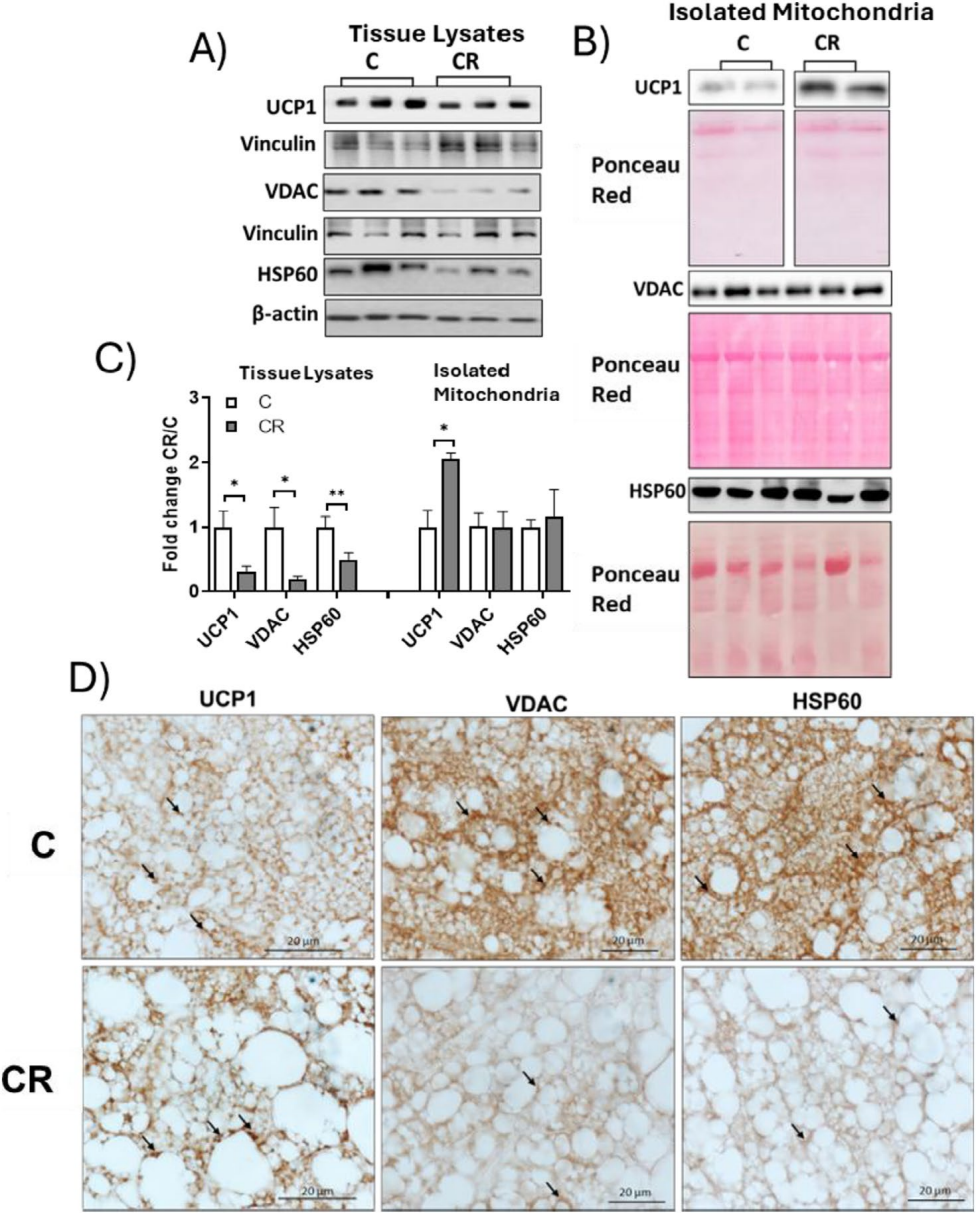
Representative Western blots of UCP1, VDAC, and HSP60 detected in BAT total lysate (A) and isolated mitochondria (B) from CR and C samples. Vinculin or Ponceau staining was used as a loading control. Each lane contained 25 μg of protein of sample from a single rat. Histograms represent quantification of the western blot data, which were normalized to the value obtained for C animals, set as 1 (C). Values represent the mean ± SEM of 4 different rats. *p < 0.05, **p < 0.01 versus C. Representative iBAT sections stained with immunohistochemistry for UCP1, VDAC, and HSP60 (D). Arrows indicate immunoreactive areas where mitochondria localize. Magnification used: 100X; scale bar applied: 10 μm.

Regarding UCP1, proteomic analysis (Figure 2A) and Western blotting (Figure 6) indicated that its levels were downregulated by CR in BAT lysate (Figure 6A), while an increase in the protein was observed when examining isolated mitochondria (Figure 6B). Furthermore, iBAT staining with UCP1 evidenced that in BAT from CR animals there was higher immunopositivity in some paucilocular cells where localized mitochondria (Figure 6D) (arrows), in agreement with WB analyses conducted in mitochondrial enriched fraction.

The observation that CR effectively increased the levels of SDHB, UQCRC2, MTCO1, ATP5A, and UCP1 in isolated mitochondria, while simultaneously decreasing their abundance in whole tissue lysates, strongly suggests a reduction in total mitochondrial content within BAT of CR‐treated rats. To clarify this possibility, we evaluated the levels of two protein markers of mitochondrial abundance, such as HSP60 and VDAC.

Indeed, immunohistochemical analyses of iBAT sections showed that CR rats exhibited weak VDAC or HSP60 staining compared to the Control group (Figure 6D, VDAC‐HSP60, black arrows). These findings align with Western blot data, which indicate a reduction in VDAC and HSP60 levels (−48% and −50%, respectively, in CR vs. C) in total tissue lysate (Figure 6A,C). Interestingly, when detected in isolated mitochondria, the levels of HSP60 and VDAC remained unaffected by CR treatment (Figure 6B,C). These data further support the conclusion that CR reduces iBAT mitochondrial content.

**FIGURE 7:**
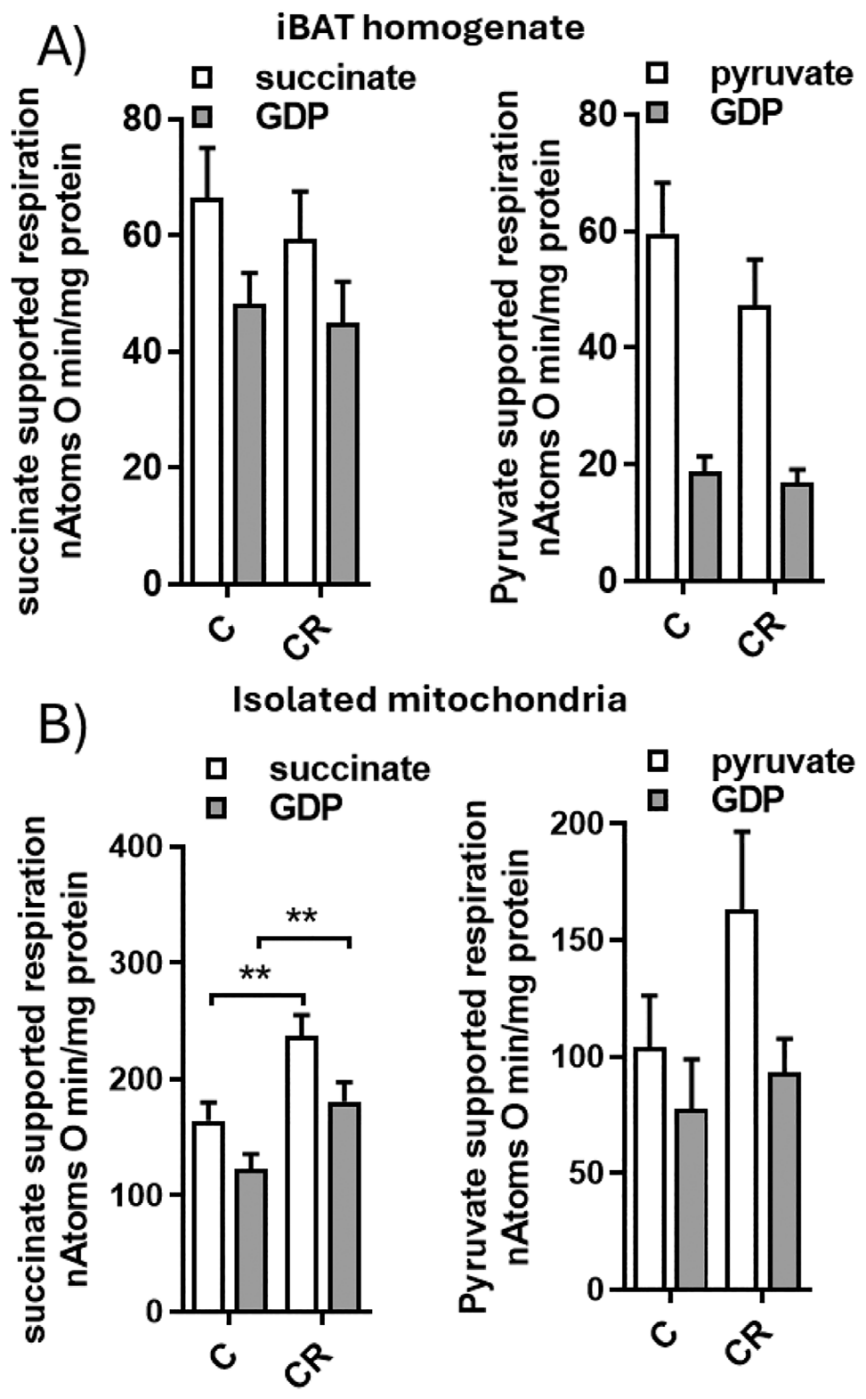
Effect of mild short‐term CR on mitochondrial respiration rate observed in the whole homogenate (A) or in isolated mitochondria (B). Respiration was measured using succinate (+rotenone) or pyruvate (+malate) as substrates, both in the absence and presence of GDP. Values represent the mean ± SEM of 6–8 different rats. **p < 0.01 versus C**.

We then measured mitochondrial respiration rates in the whole fatty acid‐deprived iBAT homogenate and isolated mitochondria. This choice is fundamental since measuring respiration rate in iBAT homogenates provides a powerful readout of both mitochondrial content and functionality, whereas in isolated mitochondria, it reveals only their intrinsic functional capacity (Figure 7). Two different respiratory substrates were used, namely pyruvate plus malate and succinate plus rotenone, which involve complex I‐ and complex II‐linked respiratory pathways, respectively. Respiration was detected both in the presence and absence of GDP, one of the inhibitors of UCP1 [52].

Indeed, when detected in the basal condition (i.e., in the presence of the substrate alone), the control of respiration is shared between the activities of the reactions involved in substrate oxidation, among these the respiratory chain and proton leak. In the presence of GDP, the control exerted by the respiratory chain on respiration becomes more relevant due to its inhibition of UCP1‐mediated proton leak [53].

When analyzed in the entire iBAT homogenate, no significant differences in respiration rates were observed between the CR and C groups (Figure 7A), regardless of the substrate used. Adding GDP to the respiration medium, which is intended to inhibit UCP1 activity, reduced mitochondrial respiration rate; however, no differences in GDP‐inhibited respiration rate were found between the C and CR groups (Figure 7A).

When isolated mitochondria were analyzed, CR tends to increase the pyruvate‐supported respiration rate; however, the differences did not reach statistical significance (Figure 7B).

In succinate‐energized mitochondria, we observed a significant increase in respiration rate induced by CR, both when it was detected in the presence and in the absence of GDP (Figure 7B), thus suggesting an improvement in succinate‐linked (complex II)‐respiratory pathways.

### 3.5 Effect of CR on Oxidative Stress

**FIGURE 8:**
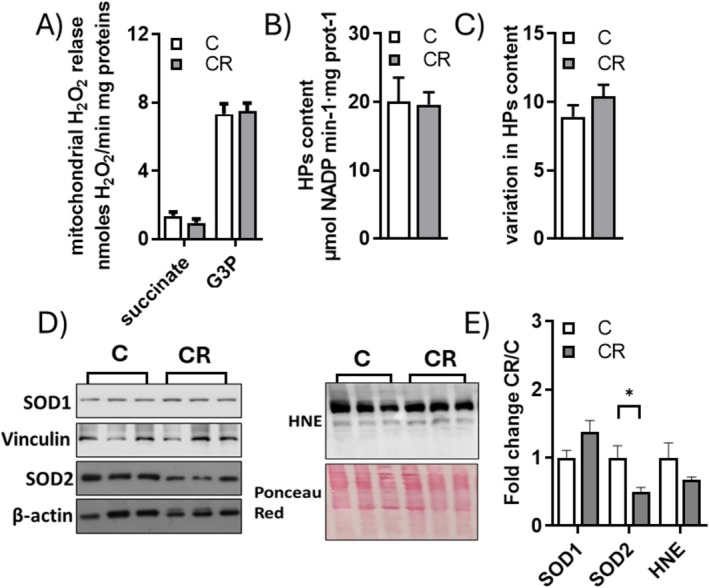
Effect of mild short‐term CR on oxidative stress. (A) Mitochondria H_2_O_2_ release, (B) lipid hydroperoxides tissue content, (C) susceptibility of tissue to an oxidative insult, (D) western blot of SOD1, SOD2, and HNE protein adduct. Each lane contained 25 μg of protein of total iBAT lysate from a single rat. (E) Histograms represent quantification of the western blot data, which were normalized to the value obtained for C animals, set as 1. Values are the mean ± SEM of 6 or 8 different rats *p < 0.05 s C.

To assess whether CR could influence oxidative stress in iBAT, we examined some markers of oxidative stress and lipid peroxidation, such as the mitochondria's ability to release H_2_O_2_ (as an index of mitochondrial superoxide formation) (Figure 8A), tissue lipid hydroperoxides levels (Figure 8B), 4‐hydroxynonenal (HNE) protein adducts levels (Figure 8C), the susceptibility of BAT to oxidative stress, as well as superoxide dismutase 1 and 2 protein levels (SOD1 and SOD2).

The ability of isolated mitochondria to release H_2_O_2_, when energized with either succinate or glycerol–3‐phosphate (G3P), was unaffected by CR. Additionally, no changes were observed in tissue’ lipid hydroperoxide levels or HNE protein adducts between the C and CR samples. Similarly, the response of BAT to an oxidative insult was not altered by CR. CR did not influence SOD1 levels but down‐regulated SOD2 levels.

### 3.6 CR Affects the Activity of Glycerol‐3‐Phosphate Shuttle Components

**FIGURE 9:**
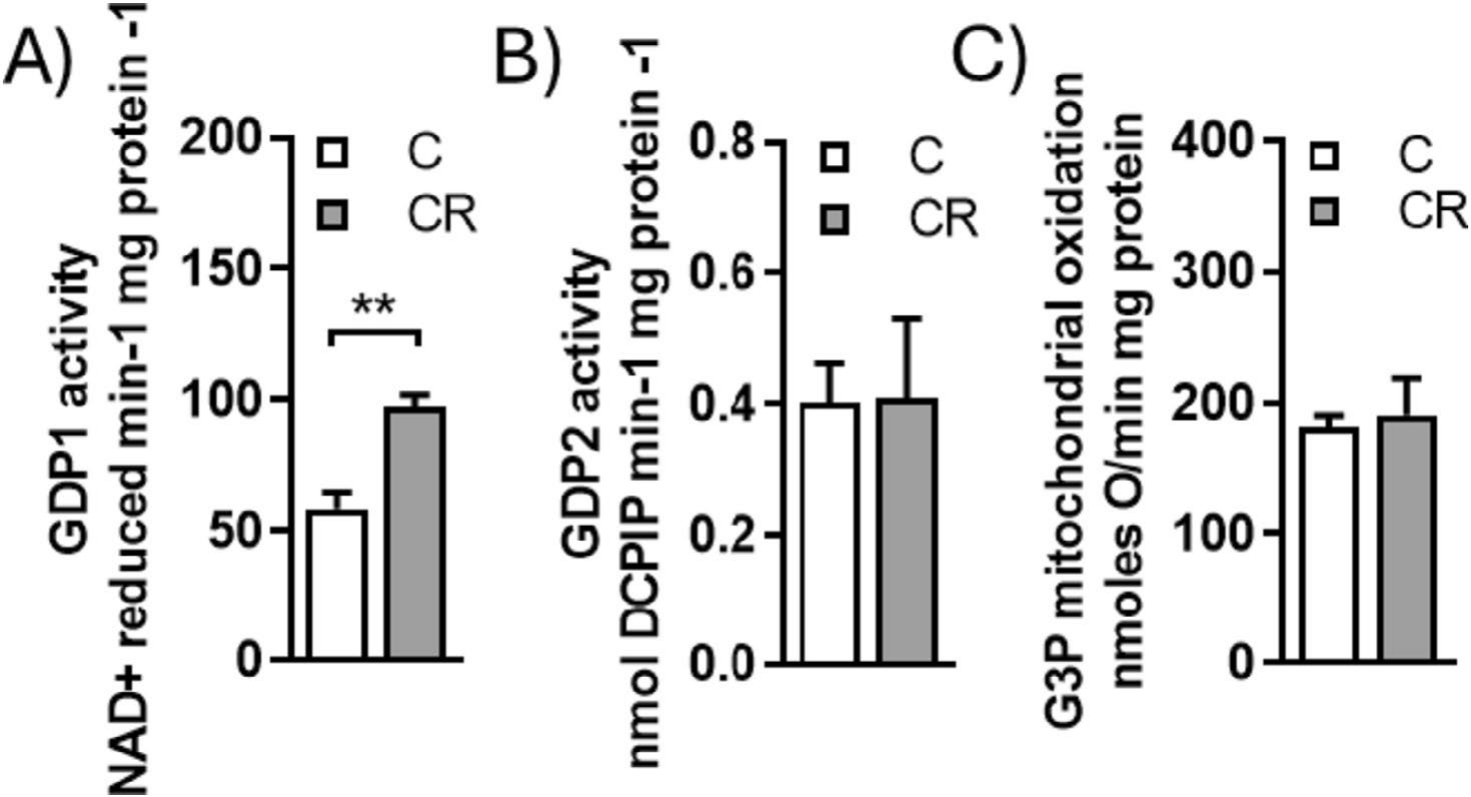
Effect of mild short‐term CR on components of glycerol phosphate shuttle. (A) Activity of cytosolic Glycerol‐3‐phosphate dehydrogenase (GPD1), (B) activity of mitochondrial glycerol‐3‐phosphate dehydrogenase (GPD2), (C) glycerol‐3‐phosphate mitochondrial oxidation. Values are the mean ± SEM of 4 different rats. **p < 0.01 versus C.

The G3P shuttle is a metabolic hub, linking glycolysis, lipid synthesis, and oxidative phosphorylation [54]. In the G3P shuttle, cytosolic glycerol‐3‐phosphate dehydrogenase‐1 (GPD1) converts dihydroxyacetone phosphate (DHAP) into G3P by reducing NAD^+^ to NADH. G3P diffuses into the mitochondrial intermembrane space and is oxidized by mitochondrial glycerol‐3‐phosphate dehydrogenase‐2 (GPD2), which, by transferring electrons to the coenzyme Q, acts as a non‐conventional component of the electron transport chain (ETC). Therefore, we assessed the activities of GPD1 and GPD2 and the ability of mitochondria to oxidize G3P. CR significantly enhanced GPD1 activity (Figure 9A) without affecting GPD2 activity (Figure 9B); furthermore, CR did not affect the ability of isolated mitochondria to use G3P as a respiratory substrate (Figure 9C).

### 3.7 CR Affects the Levels of Enzymes Involved in Amino Acid Synthesis and the BCAAs Degradative Pathway, Without Affecting Their Tissue and Serum Levels

**FIGURE 10:**
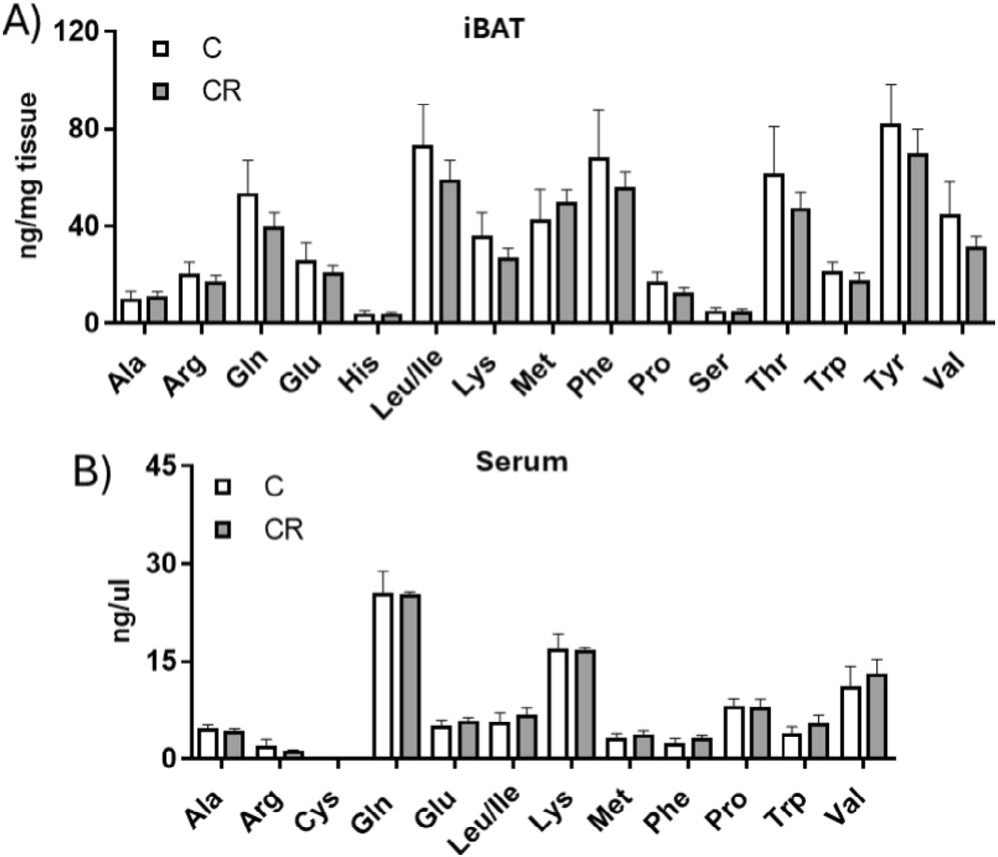
Effect of mild short‐term CR on amino acid levels detected in iBAT (A) and sera (B) extracts. Values represent the mean ± SEM of 4 different rats.

Since KEGG enrichment pathway analysis indicated that more down‐regulated proteins, identified through a proteomic approach, belong to the BCAAs degradation pathway, we explored the potential effects of CR treatment on tissue and serum BCAAs levels. Interestingly, through targeted metabolomics, we found no significant changes in the levels of BCAAs and other amino acids in both iBAT tissue and serum between the control and CR groups (Figure 10A,B).

### 3.8 CR Influences the Collagen Deposition in the Perivascular Area and Intermediate Filaments Proteins

Among CR's most upregulated proteins detected in the iBAT lysate are lumican and decorin (Figure 2A), i.e., two extracellular matrix proteins involved in forming collagen fibrils; thus, we evaluated whether CR could influence collagen deposition.

**FIGURE 11:**
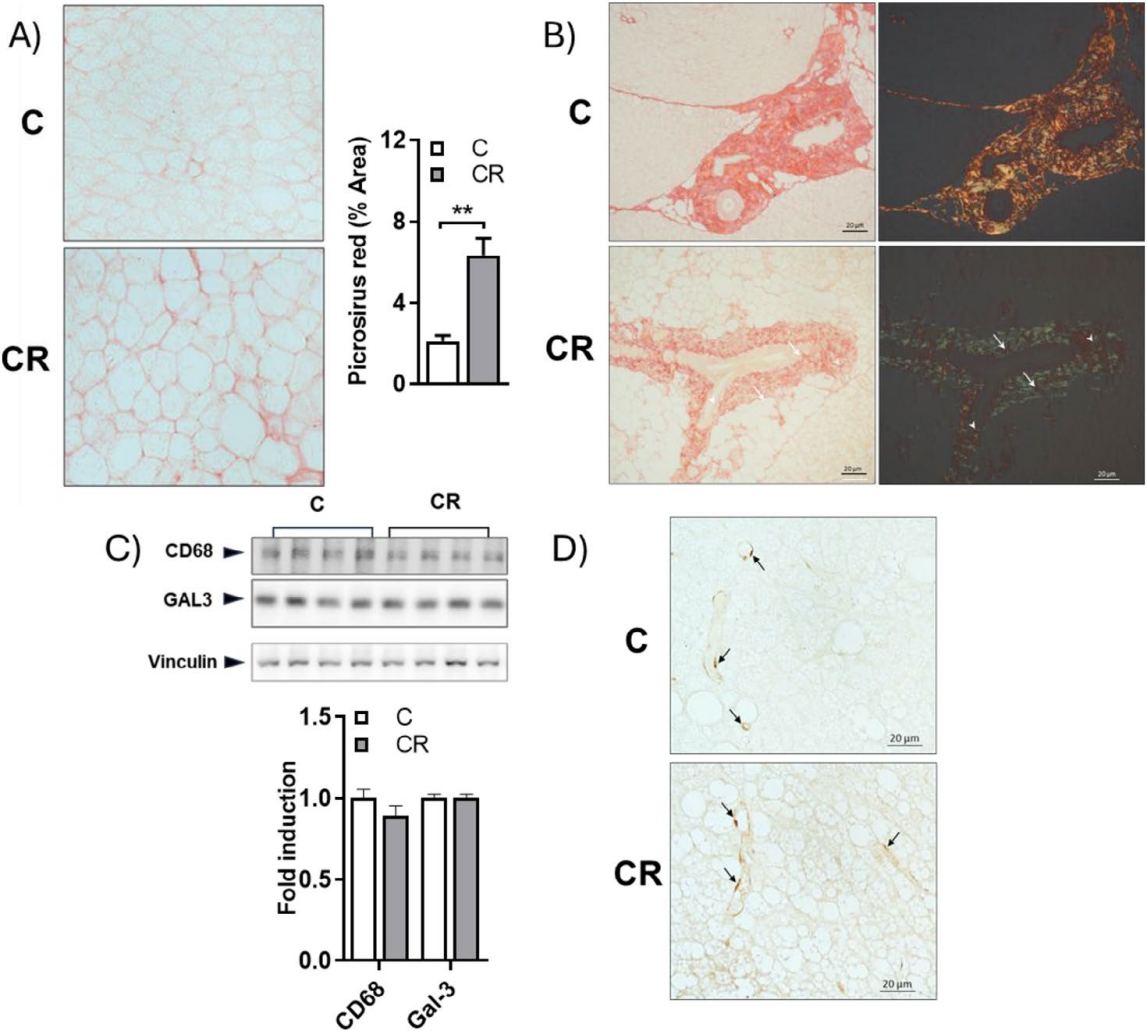
Effect of mild short‐term CR on collagen fibers and macrophage presence in iBAT. (A) Histological section of iBAT stained with picrosirius red observed under optical light microscopy. Histograms represent the quantification of Picrosirius red stained area. (B) Histological section of iBAT stained with picrosirius red observed polarized light microscopy. Arrows indicate the newly synthesized collagen III thin fibers, that appears green; arrowhead evidence yellow/orange or red collagen fibers (collagen I, thick fibers). Magnifications used: 10X or 20X; scale bar applied: 20 μm. (C) Representative Western blotting analysis of CD68 and galectin (Gal3) protein levels. Each lane contained 30 μg of protein of total iBAT lysate from a single rat. Vinculin was used as the loading control. Histograms show quantification of the signals expressed relative to the value obtained from the control, and represent means ± SEM for 4 different rats. (D) Histological section of iBAT stained with immunohistochemistry for CD68. Arrows indicate macrophages localization in the tissue. Magnification used: 40X; scale bar applied: 20 μm.

For this purpose, we used histological sections of iBAT stained with picrosirius red and observed them under optical and polarized light microscopy (Figure 11A,B respectively). The quantification of collagens stained red in the intercellular spaces in iBAT indicates a significant increase in peri‐adipocyte collagen accumulation in the CR group. In addition, when analyzed by plane‐polarized light, the images of the stained sections from CR rats indicate variations in collagen fibers appearing green (newly synthesized collagen III thin fibers, arrowhead), yellow/orange or red (collagen I thick fibers, white arrow), especially around the perivascular area. The iBAT section from CR rats showed an enhanced green signal and lower red signal compared to controls, thus suggesting that CR induces the formation of new thin fibers (Figure 11A).

**FIGURE 12:**
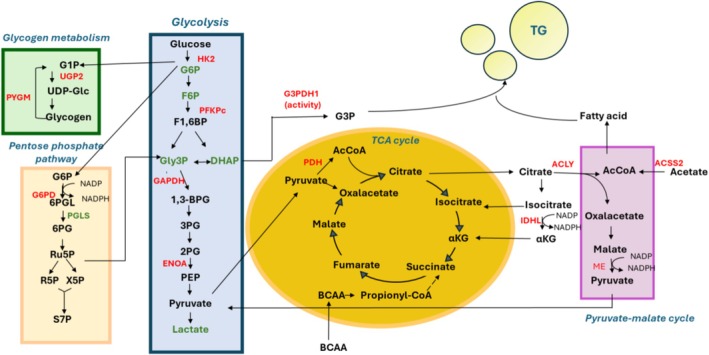
Scheme depicting the metabolic pathways influenced by a mild and short‐term CR. The enzymes upregulated by mild short‐term CR are colored in red, the metabolites and the enzymes downregulated are colored in green. 1,3‐PG, 1,3‐Phosphoglycerate; 2PG, 2‐Phosphoglycerate; 3PG, 3‐Phosphoglycerate; 6PG, 6‐Phosphogluconate; 6PGL, 6‐Phosphoglucono‐δ‐lactone; AcCoA, Acetyl‐CoA; BCAA, Branched chain amminoacid; DHAP, Dihydroxyacetone phosphate; F1,6BP, Fructose‐1,6‐bisphosphate; F6P, Fructose‐6‐phosphate; G1P, Glucose‐1‐phosphate; G3P, Alpha glicero phosphate; G6P, Glucose‐6‐phosphate; Gly3P, Glyceraldehyde‐3‐phosphate; R5P, Ribose‐5‐phosphate; Ru5P, Ribulose‐5‐phosphate; S7P. Sedoheptulose‐7‐phosphate; TG, Triglycerides; UDP‐Glc, Uridine Diphosphate Glucose; XuP, Xylulose‐5‐phosphate; α‐KG, Alpha ketoglutarate.

Since fibrillogenesis could be associated with macrophage infiltration within the tissue, we evaluated some markers of their presence, such as Galectin‐3 (Gal‐3) and CD68, in BAT. Western blot analysis and immunohistochemistry revealed no changes in the levels of these proteins between CR and C rats (Figure 11C,D, respectively), thus excluding macrophage infiltration into the tissue. Indeed, immunoreactive cells were found localized in vessels and not in adipose tissue parenchyma around adipocytes (Figure 12D, arrows).

## 4 Discussion

The manipulation in BAT physiology and CR has been reported as an important intervention able to improve metabolic health. The current study highlights that CR, even mild and applied for just 2 weeks, induces coordinated metabolic and structural changes in the iBAT, offering further insight into the metabolic processes modulated by CR.

Our data indicate that in iBAT, mild short‐term CR promotes TG accumulation, as shown by increased LD size; this effect is consistent with previous studies on rat models, which applied a 30% CR over six‐month periods [31].

In BAT, support for thermogenesis through glucose metabolism is limited, whereas glucose is crucial for providing fuel for de novo lipogenesis [12]. Additionally, the carbohydrate storage as glycogen and its metabolism play a vital role in BAT physiology and LD deposition [55]. Our findings indicate that a mild short‐term CR influences glucose metabolism by affecting glycogen turnover and glucose oxidation. Tissue staining reveals reduced glycogen levels, suggesting that iBAT adapts by supplying substrates for glycolysis (see schema Figure 12, green box). Additionally, iBAT from CR treated rats shows increased levels of glycolytic enzymes, stable tissue glucose, and decreased glycolytic intermediates, indicating enhanced glycolytic flux, which fuels lipogenesis (Figure 12, blue box). The capacity of mild short‐term CR to raise glycolytic‐related protein levels is consistent with studies performed in mice subjected to CR 30% for 8 weeks, which reported increased mRNA expression of proteins involved in this process [34].

It is known that to sustain a high glycolysis rate, NADH produced by GAPDH has to be promptly oxidized to regenerate NAD^+^ [42, 56]; in agreement with this concept, we showed that mild short‐term CR facilitates this regeneration since it enhances the activity of the cytosolic GPD1, a component of the glycerol‐3‐phosphate shuttle, which oxidizes NADH and converts glycolysis‐derived DHAP into G3P. The latter is essential for TAG synthesis or used as a mitochondrial respiratory substrate by GPD2. Our data also suggest that G3P formed by GPD1 activity serves as the structural backbone for TAG synthesis [54, 57], rather than being used as a mitochondrial respiratory substrate, since CR does not affect GPD2 activity and the mitochondrial ability to utilize G3P as a respiratory substrate.

Mild short‐term CR also promotes the import of pyruvate into the mitochondria and its decarboxylation to Acetyl‐CoA, as indicated by an enhancement in the mitochondrial pyruvate carrier subunit MCP2 and PDH levels. It is well recognized that within the matrix, Acetyl‐CoA reacts with oxaloacetate to produce citrate (see brown box Figure 12), which can either enter the tricarboxylic acid (TCA) cycle or exit the mitochondria to reach the cytosol, where it can be converted back into acetyl‐CoA through the activity of ACLY, a key enzyme linking glucose and fatty acid metabolism [58]. Our data suggest that mild short‐term CR increases the levels of ACLY and ACSS2; the latter is unique in its ability to produce Acetyl‐CoA from acetate for lipid biosynthesis and complements ACLY in the production of Acetyl‐CoA via the pyruvate route [59]. The enhancement in ACLY levels aligns with data from the literature, where 30% CR was applied for 30 days [31].

Mild short‐term CR is also effective in enhancing the levels of cytosolic enzymes catalyzing the production of NADPH, which is crucial for the de novo lipogenesis process, such as G6PD, ME, and IDH (see schema Figure 12). In addition to generating NADPH, G6PD also plays a key role in the pentose phosphate pathway (PPP). However, despite the increase in G6PD protein levels, the concentration of the metabolite ribose‐5‐phosphate, one of the principal products of PPP, is not significantly affected by mild short‐term CR (Figure 12, yellow box). This may be due to CR‐induced 6‐phosphogluconolactonase (PGLS) downregulation, which catalyzes the second step of the PPP, thereby suggesting a bottleneck at this step, which limits the PPP flow. Thus, the enhanced G6PD levels seem to be more functional to de novo lipogenesis rather than to enhancing PPP flow.

Regarding LD enlargement, more proteins up‐regulated by mild short‐term CR are involved in this process, among these are ACSL5, AKR1C15/17‐βHSD, and RAB1B. ACSL5 catalyzes the formation of fatty acyl‐CoAs from long‐chain fatty acids (C16–C20), which can be used for TG synthesis [60]; RAB1B targets diacylglycerol acyltransferase from the endoplasmic reticulum to the LDs, thus promoting their growth [61]; AKR1C15 is an LD‐associated protein involved in LD dynamics, capable of preventing lipolysis [62].

KEGG analysis of dysregulated proteins identified by proteomic approach shows that one of the pathways mainly enriched in proteins down‐regulated by mild short‐term CR is BCAAs degradation. However, we do not detect significant changes in BCAA and other amino acids levels both in iBAT and serum samples. On a speculative level, it is possible to suggest that the reduction in enzymes involved in the BCAA degradation could be functional to their use for lipogenesis and the synthesis of branched‐chain fatty acids. Further detailed studies, specifically designed to investigate metabolic flux, will be required to clarify this aspect.

Regarding iBAT thermogenesis, some studies present in the literature indicate that in mice and rats, long‐term CR was effective in declining it [31, 36]. At first glance, the reduction in mitochondrial respiratory complexes subunits, UCP1 and mitochondrial content markers, as well as by the down‐regulation of oxidative phosphorylation and thermogenesis pathway, revealed by KEGG analysis, suggests that mild short‐term CR also reduces iBAT thermogenic capacity by reducing mitochondrial content. However, these results apparently contrast with the unchanged iBAT whole homogenate mitochondrial respiration rate. Nevertheless, it should be noted that, when measured in whole homogenate, mitochondrial respiration rate reflects both mitochondrial content and functionality, whereas its evaluation in isolated mitochondria provides information only on the functional capacity of the organelles. In this context, we demonstrated that mild short‐term CR increases the mitochondrial respiration rates in isolated organelles, by enhancing respiratory chain subunits levels, respiratory chain activity, and UCP1. Therefore, overall, our data suggest that improved mitochondrial functionality compensates for the CR‐driven decrease in mitochondrial content, maintaining iBAT thermogenic capacity unchanged.

Increases in mitochondrial oxygen consumption or mitochondrial membrane potential, driven by electron transport chain activity, can lead to enhanced ROS production [63]. Our data indicate that, although mild short‐term CR increases mitochondrial respiration rate, mitochondrial H_2_O_2_ release (used as an index of mitochondrial ROS production) remains unchanged. It is plausible that the rise in mitochondrial UCP1 levels, which dissipate proton motive force across the inner mitochondrial membrane and reduce membrane potential [64], could compensate for the higher activity of the respiratory chain and thereby maintain ROS at a constant level. We also observed a decrease in SOD_2_ levels in iBAT lysate, which, given its mitochondrial localization, aligns with the reduction in tissue mitochondrial content induced by mild short‐term CR.

It is important to note that the mitochondrial isolation protocol used in this study does not allow for the separation of PDM and CM subpopulations, as our aim was to gain a comprehensive representation of the entire iBAT mitochondrial population. Since in iBAT, mitochondria attached to LD have been shown to contribute to LD expansion and the two mitochondrial populations have different bioenergetics capacity [15], further studies focusing on separate PDM and CM subpopulations will be needed to clarify their distinct bioenergetics contributions to BAT function under mild short‐term CR conditions.

In iBAT structural and morphological remodeling, involving cytoskeleton and the extracellular matrix (ECM), are necessary to support new functional demand and to accommodate larger LDs. ECM remodeling has been described in both physiological and pathological conditions, such as cold exposure and obesity; it has been considered essential to sustain BAT activation, with collagen network fibers maintaining tissue integrity and elasticity and allowing BAT to expand and contract during thermogenesis [65]. Our histological data suggest that mild short‐term CR induces EMC remodeling involving the collagen network, since it enhances collagen deposition in the preadipocyte area and collagen III deposition in perivascular areas. Increased levels of decorin and lumican, two proteoglycan proteins involved in collagen fibrillogenesis [66, 67], may have played a significant role in this process. In addition, we found no changes in the levels of macrophage markers in the tissue, thus leaving out the occurrence of inflammation and fibrosis processes associated with fibrillogenesis.

Intermediate filament (IFs) networks may reorganize to accommodate larger LDs or redistribute tension within the cell [68] and our proteomic analysis indicates that some proteins forming IFs, such as KRT10, KRT1, and AABR07001416.1, are upregulated by mild short‐term CR by more than 2‐fold (Figure 2).

Our study has some limitations. Our analyses are limited to male rats; thus, further studies will be needed to evaluate the potential sexually dimorphic response of BAT to mild short‐term CR and to determine whether the patterns observed in rats are similarly found in mice. In addition, since for proteomic study pooling the samples does not allow us to consider the variability between biological samples, for those proteins whose levels were not validated by western blot, there is a possibility that an outlier sample drives their variation.

Our study also opens new opportunities for further studies to assess how long changes induced by mild short‐term CR in BAT persist after its discontinuation and whether the adaptations observed within 2 weeks are maintained with more prolonged treatments.

## 5 Conclusions

Taken together, the data indicate that CR, even when mild and applied for only 2 weeks, is able to profoundly influence iBAT metabolism and morphology, which may represent a key mechanism underlying its broad systemic effects, offering new insights into the role of CR in maintaining overall health.

Our data allow to suggest that TG accumulation within adipocytes, induced by mild short‐term CR, may serve as an energy reserve, enhancing iBAT flexibility in conditions that require increased heat production during energy scarcity. This is particularly relevant when circulating free fatty acids from WAT lipolysis and triglyceride‐rich lipoproteins are utilized to sustain the energy requirements of tissues other than iBAT. Moreover, CR‐induced mitochondrial content reduction, associated with increased mitochondrial respiration, provides advantages in terms of cellular energetic costs for maintaining thermogenesis under energy restriction.

Overall, considering the limited long‐term adherence typically associated with calorie‐restricted diets, the finding that even mild short‐term CR induces metabolic adaptations in iBAT highlights a potential impact for translational research.

## Author Contributions

A. Lombardi, P. de Lange, L. Lionetti, M. Moreno conceived and designed the research; G. Panico, V. Migliaccio, *G. pinto* , A. Iliano, G. Fasciolo, M. Coletta performed the research and acquired the data, A. Amoresano, L. Lionetti, P. Venditti, V. Migliaccio, *G. pinto* , P. de Lange, A. Lombardi analyzed and interpreted the data. A. Lombardi, G. Panico, *G. pinto* were involved in drafting manuscript. All the authors revised the manuscript.

## Funding

This work was supported by Ministero dell'Istruzione, dell'Università e della Ricerca (MUR), P2022Z2JS3.

## Conflicts of Interest

The authors declare no conflicts of interest.

## Supporting information


**Data S1:** Supporting Information.

## Data Availability

The data that support the findings of this study are available in the Results and Supporting Information S1 of this article.
